# Biomechanical Characterization of Retinal Pigment Epitheliums Derived from hPSCs Using Atomic Force Microscopy

**DOI:** 10.1007/s12015-024-10717-3

**Published:** 2024-04-16

**Authors:** Elise Herardot, Maxime Liboz, Guillaume Lamour, Michel Malo, Alexandra Plancheron, Walter Habeler, Camille Geiger, Elie Frank, Clément Campillo, Christelle Monville, Karim Ben M’Barek

**Affiliations:** 1 Université Paris-Saclay, Univ Evry, INSERM, IStem, UMR861, 91100 Corbeil-Essonnes, France; 2 Université Paris-Saclay, Univ Evry, CY Cergy Paris Université, CNRS, LAMBE, 91025 Evry-Courcouronnes, France; 3 IStem, CECS, 91100 Corbeil-Essonnes, France; 4 Institut Universitaire de France (IUF), Paris, France

**Keywords:** Retinal pigment epithelium, Human pluripotent stem cells, Atomic force microscopy, Nanomechanic, Retinitis pigmentosa, Cell therapy

## Abstract

The retinal pigment epithelium (RPE), a multifunctional cell monolayer located at the back of the eye, plays a crucial role in the survival and homeostasis of photoreceptors. Dysfunction or death of RPE cells leads to retinal degeneration and subsequent vision loss, such as in Age-related macular degeneration and some forms of Retinitis Pigmentosa. Therefore, regenerative medicine that aims to replace RPE cells by new cells obtained from the differentiation of human pluripotent stem cells, is the focus of intensive research. However, despite their critical interest in therapy, there is a lack of biomechanical RPE surface description. Such biomechanical properties are tightly related to their functions. Herein, we used atomic force microscopy (AFM) to analyze both the structural and mechanical properties of RPEs obtained from four cell lines and at different stages of epithelial formation. To characterize epitheliums, we used apical markers in immunofluorescence and showed the increase of transepithelial resistance, as well as the ability to secrete cytokines with an apico-basal polarity. Then, we used AFM to scan the apical surface of living or fixed RPE cells. We show that RPE monolayers underwent softening of apical cell center as well as stiffening of cell borders over epithelial formation. We also observed apical protrusions that depend on actin network, suggesting the formation of microvilli at the surface of RPE epitheliums. These RPE cell characteristics are essential for their functions into the retina and AFM studies may improve the characterization of the RPE epithelium suitable for cell therapy.

**Graphical Abstract:**

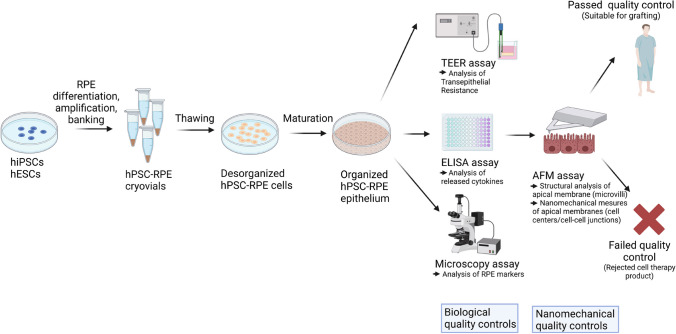

**Supplementary Information:**

The online version contains supplementary material available at 10.1007/s12015-024-10717-3.

## Introduction

Retinal degenerative diseases engender the death of photoreceptors, which are the light sensing cells essential for vision. These diseases may be caused by genetic alterations or dysfunctions of photoreceptors and/or retinal pigment epithelium cells (RPE cells), such as in age-related macular degeneration (AMD) and retinitis pigmentosa (RP) [1–3]. To date, no effective treatment has been proposed for the majority of patients. RPE cell dysfunctions participate to AMD pathology and represent 5% of RP cases [4]. Hence, replacement of dysfunctional or dead RPE cells by new cells generated in vitro is an attractive strategy to restore RPE cell functions and prevent photoreceptor loss.

Current cell therapy approaches rely on the differentiation of human pluripotent stem cells (hPSCs) into RPE cells. Several laboratories as well as our group have developed such protocols to generate RPE cells, to characterize these cells and set up transplantation strategies to deliver the cell therapy in rodents and primates [4, 5]. First-in-man clinical trials aiming to evaluate safety of the cell therapy were launched with some early positive safety results already published [6–9].

These first generations of cell therapies still need to be further optimized to be more effective (through improved epithelial organization prior to implantation), to be scaled-up to address the target patient population and to identify controls informative of the quality of the cells to be used for cell therapy. Indeed, as living biological products, RPE cells have more variability compared to chemicals with well-characterized properties. Thus, understanding RPE cell structure and normal functions appears necessary for the efficient development of a cell therapy that will produce donor cells with controlled characteristics. For example, Ye and collaborators recently proposed a machine-learning-based model to predict cell quality, based on microscopic images of epithelium obtained from human induced pluripotent stem cells (hiPSC)-derived RPE cells [10].

Here, we pursue this effort of characterizing hPSC-RPE sheets by measuring the mechanical properties of RPE cells. A few studies have addressed the mechanics of adult RPE cells [11–13]; yet, none has investigated the mechanics of cells derived from hPSCs. Mechanical properties are critical to the epithelial functions of RPE cells, that are an important part of the blood-retina barrier (BRB) separating the choriocapillaris from the neural retina [11]. Indeed, tight junctions interconnecting RPE cells ensure the sealing of the BRB by preventing paracellular permeability. BRB disruption may have irreversible consequences to neural retina as observed in several diseases such as AMD, inflammation or metabolic diseases [14]. In addition, mechanical properties of RPE cells can be altered by hyperosmotic stress, a phenomenon observed in macular edema [11]. Thus, external cues could affect the mechanical properties of cells forming an epithelium. Changes in the cytoskeleton organization and subsequent mechanical properties can be viewed as an early and sensitive indicator of sub-lethal stress [12, 15, 16]. Moreover, several human diseases are correlated with abnormal cell stiffening or softening [17, 18]. Therefore, characterizing hPSC-RPE cell mechanical properties will help setting up quality controls for cell therapy products.

In this study, we differentiated three hPSC lines into RPE cells to evaluate their quality and the functions of newly formed epitheliums. Based on these characteristics, we compared their performance according to the cell lines. We then evaluated mechanical characteristics at a nanometric scale of these hPSC-RPE cells using an atomic force microscope (AFM). We observed that nanomechanical properties of RPE cells were modified after epithelial formation. Our results lay the foundation of a better understanding of epithelial mechanical characteristics of RPE epitheliums derived from hPSCs.

## Results

### RPE Cells Differentiated from hPSCs Form a Cohesive Epithelium Following In Vitro Cell Culture

**Fig. 1:**
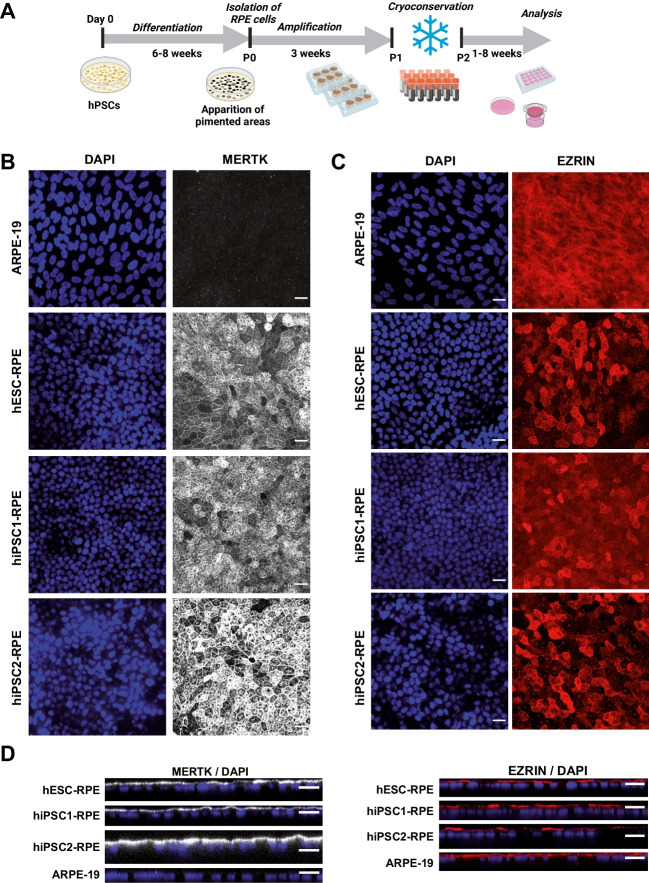
RPE cells differentiated from hPSCs form a cohesive and mature epithelium. (**A**) Timeline of the differentiation protocol to produce RPE cells from hPSCs. This protocol is based on a spontaneous differentiation protocol. (B-C) Confocal images of 2-month old RPE epithelium from the four cell lines, after immunostaining with antibodies against MERTK (**B**) and EZRIN (**C**). Images correspond to maximal projections of z stacks. (**D**) Confocal images corresponding to maximal projections in zx plans from stacks of images on B-C. Nuclei were counterstained with DAPI. Scale bar = 20 µm

**Fig. 2:**
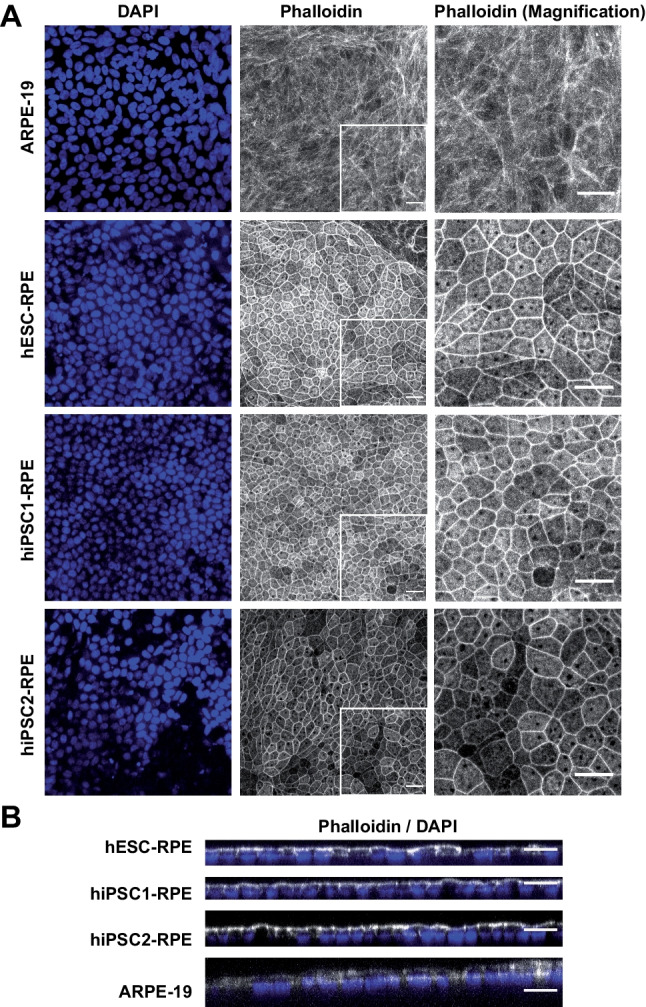
Organization of Actin microfilaments in RPE epitheliums. (**A**) Confocal images of 2-month old RPE epitheliums that were labelled with phalloidin-A647 to visualized actin networks and counterstained with DAPI. (**B**) Stack of images in A were resliced in the zx axis (maximal projections). Scale bar = 20 µm

RPE cells can be obtained from the differentiation of both human embryonic stem cells (hESCs) and human induced pluripotent stem cells (hiPSCs), with equivalent visual outcomes following transplantation in vivo [19] and both cell sources are currently evaluated in clinical trials [20]. Therefore, we differentiated three hPSCs (1 hESC line and 2 hiPSC lines) into RPE cells using an established protocol [21]. Briefly, hPSCs were seeded and grown to confluence before withdrawal of fibroblast growth factor 2 (FGF2; used to maintain the pluripotency, Fig. 1A). After 6–8 weeks of culture, pigmented patches appeared in culture dishes and were collected manually using a scalpel. We further amplified these patches for three additional weeks and froze cells in cryovials for future use on demand. The different cell lines were thawed and grown in culture dishes for two months to form an epithelium. hPSC-RPE epitheliums from the three lines exhibited a classical RPE cobblestone morphology and formed an epithelial monolayer. Immunofluorescence assays revealed the polarization of key epithelial markers (Fig. 1B-D), which localized at the apical side of monolayers. First, we labelled MER proto-oncogene tyrosine-protein kinase (MERTK) protein (an apical marker) that is involved in phagocytosis function of RPE cells. Second, EZRIN indicated the presence of microvilli on top of RPE cells [22, 23]. We also observed actin microfilaments at the apical side of the cells, with a clear local enrichment at cell–cell borders (Fig. 2A-B). These data suggest the formation of a cohesive epithelium, with tight junctions for the three hPSC-RPE lines. We also evaluated the formation of an epithelial monolayer from an adult RPE line (ARPE-19 line) that is classically used as an immortal adult RPE cell line. As observed in other studies [24, 25], ARPE-19 cells have lost some typical RPE characteristics such as pigmentation and do not form a complete mature epithelium. Indeed, some epithelial markers are missing, such as MERTK or cortical actin in the apical side (Figs. 1 and 2). Nevertheless, we used this adult RPE line as a RPE line lacking some crucial maturation markers for comparison with hPSC-RPE cell lines.

### Functionality of hPSC-RPE Increases Upon Epithelial Formation In Vitro

**Fig. 3:**
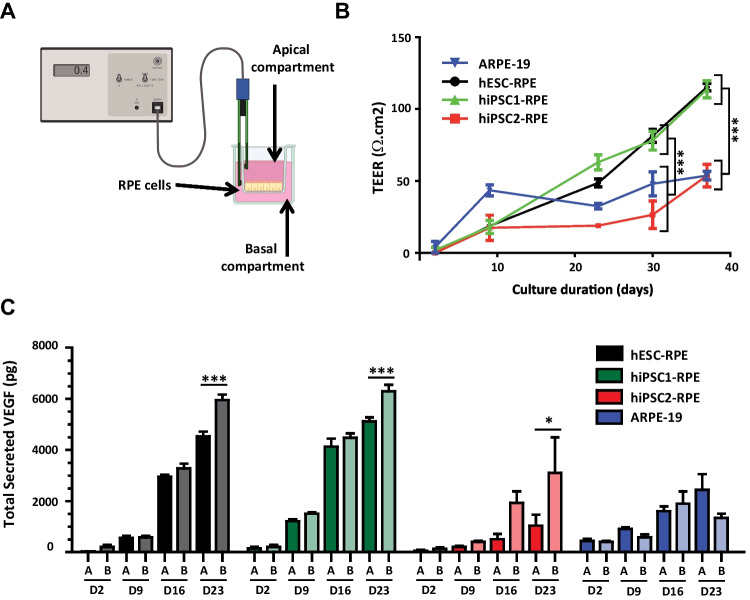
Increased functionality of RPE epitheliums upon maturation in vitro. (**A**) Drawing illustrating the system used to measure the TEER in vitro. (**B**) Quantification of the TEER according to the time of maturation in culture for the four RPE cell lines. (**C**) Quantification of the polarized secretion of VEGF by the four RPE cell lines during three weeks in cultures via ELISA assays. A: apical; B: basal. Values correspond to three different culture inserts per condition and are plotted as mean ± SEM. Significant two-way ANOVA were followed up with Bonferroni post test. *p < 0.05; ***p < 0.001

Upon epithelial formation of RPE sheets, tight junctions start to appear and the paracellular diffusion of molecules becomes limited. This barrier function, essential in the BRB can be assayed by measuring the resistance to an electrical current (transepithelial electrical resistance (TEER)) [26]. We evaluated the rise of TEER upon epithelial formation of the four RPE lines during 37 days (Fig. 3A–B). TEER increased over time for all conditions, suggesting epithelial formation and effective barrier function. However, hESC-RPE and hiPSC1-RPE cells had a significantly higher TEER than hiPSC2-RPE and ARPE-19 cells. At D37, hESC-RPE and hiPSC1-RPE cells had a TEER of 115 and 114 Ω.cm^2^, respectively, similar to living human RPE values (150 Ω.cm25]). hiPSC2-RPE and ARPE-19 cells had a lower TEER of 54 Ω.cm^2^ (a typical low value observed in other ARPE-19 studies [24, 27]). These data suggest that the different RPE cell lines can all produce an epithelium, with varying levels of quality/performance as indicated by differences in TEER.

We then evaluated the release of vascular endothelial growth factor (VEGF), another critical function of RPE epitheliums. VEGF is released preferentially in the basal side of RPE (toward the choroid in vivo to ensure the maintenance of the choriocapillaris [28]). The four RPE cell lines were cultured on Transwell permeable supports to monitor VEGF release on apical and basal compartments over time, using VEGF ELISA assays. We observed an increased secretion of VEGF over time in all compartments for all RPE cell lines (Fig. 3C). A significantly higher basal secretion at D23 was observed for the three RPE lines derived from hPSCs, when compared to corresponding apical compartments. Such increased basal secretion was not observed with ARPE-19 cells up to 23 days. In addition, VEGF secretion amounts were different according to the RPE cell lines. Similar to TEER measures, hESC-RPE and hiPSC1-RPE cells were more performant, with higher amounts of secreted VEGF compared to hiPSC2-RPE and ARPE-19 cells. Taken together, TEER and VEGF secretion assays provide strong support for the formation of functional epitheliums in vitro from hPSC-RPE cells.

### Mechanical Properties of hPSC-RPE Cells Change Upon Epithelial Formation

**Fig. 4:**
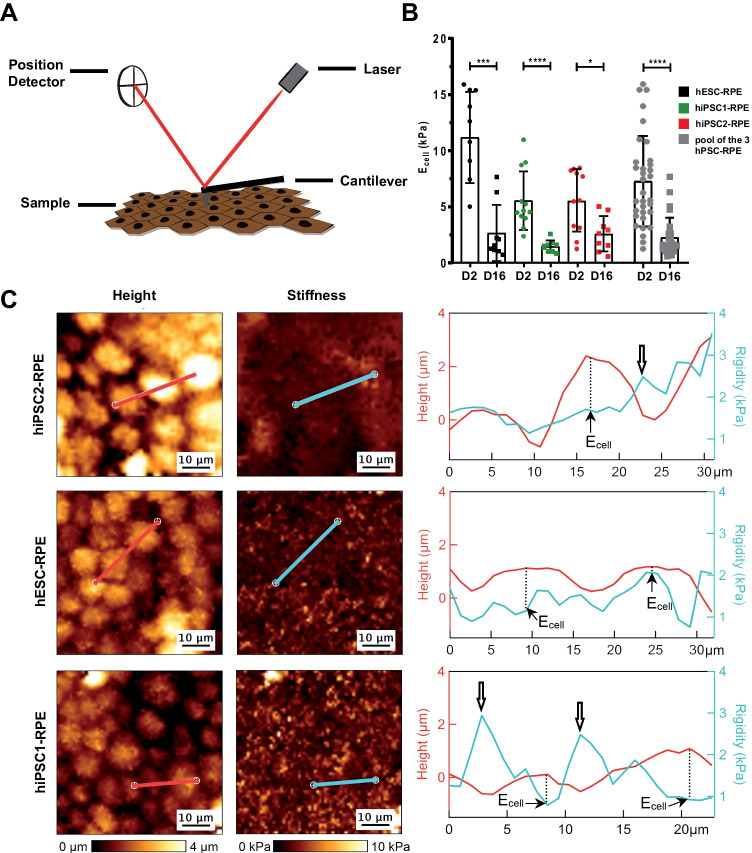
Mechanical properties of hPSC-RPE epitheliums are modified following epithelial maturation**.** (**A**) Scheme depicting the AFM principle for the recording of nanomechanical characteristics of the apical side of RPE epitheliums. (**B**) Quantification of apparent Young’s Modulus at the center of the cells (E_cell_) for the three hPSC-RPE lines at day 2 (D2) and D16. 9–12 cells were analyzed per condition. Values plotted are mean ± SD. Mann Whitney test was used for statistical comparisons. *p < 0.05; ***p < 0.001; ****p < 0.0001. (**C**) AFM topographical images (height) and corresponding elasticity maps for the three hPSC-RPE lines after a 2-week culture period. A representative line was traced in images (red and blue lines) and plotted in right panels to show the distribution of stiffness according to the cell height measured by AFM. White arrows indicate cell junctions stiffer than cell centers

To further characterize functional epitheliums, we measured mechanical properties of living RPE cells derived from hPSCs with force-indentation experiments using an atomic force microscope (AFM) (Fig. 4A). Thanks to a small tip (radius < 100 nm) interacting with samples at piconewton forces, AFM simultaneously provides nanoscale topography and mechanical measurements of soft matter materials in situ, such as living cells [29] or tissues [18] or biomimetic materials [30]. The cell stiffness is quantified by the Young’s modulus (in N.m^−2^ or pascals), and a higher or lower modulus corresponds to a stiffer or softer material, respectively.

For the three RPE cell lines derived from hPSCs, cell stiffness measured on the apical surface significantly diminished upon epithelial formation from day 2 to day 16 (Fig. 4B). During this time period, RPE cells derived from hPSCs proliferated to reach confluence from 6.4 × 10^4^ (± SEM 8.1 × 10^3^) cells per cm^2^ to 8.4 × 10^5^ (± SEM 6.8 × 10^4^) cells per cm^2^. As a result, stiffness inversely correlates with cell density and epithelial formation.

Next, we measured the stiffness of cell junctions between two cells to compare it to cell centers after 2 weeks of epithelial formation. A representative line was traced in each image and was plotted in a graph to show the height and stiffness along the cross-section corresponding to that line (Fig. 4C). In some instances, cell junctions appeared stiffer than cell centers (case of hiPSC1-RPE, bottom graph), but not all cell junctions had this specific pattern. Collectively, these results suggest that cell stiffness is an indicator of both RPE epithelial reformation and apparition of tight junctions.

### Stiffness of RPE Cells Forming an Epithelium Depends on Actin Networks

**Fig. 5:**
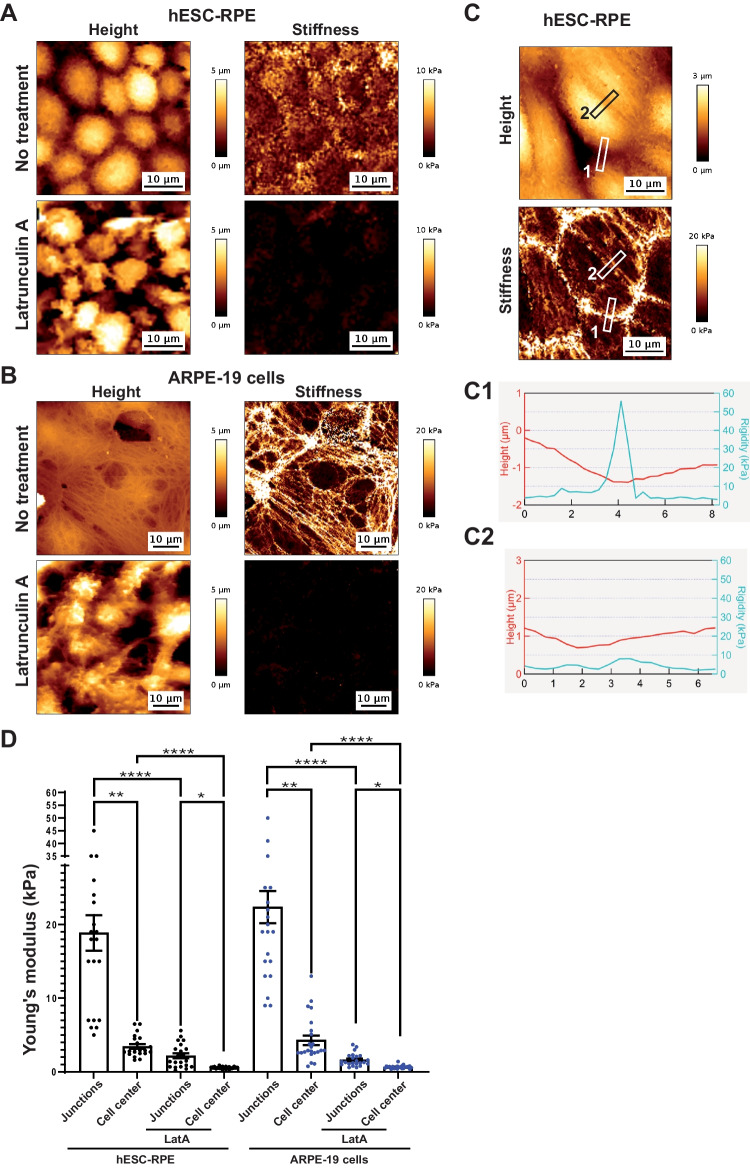
Stiffness of RPE epitheliums is dependent on actin microfilaments. (**A–B**) AFM topographical images and corresponding elasticity maps of 2-week old RPE cultures (hESC-RPE (**A**) and ARPE-19 cells (**B**)), before and after Latrunculin A treatment. (**C**) Two representative areas in hESC-RPE topographical images and corresponding elasticity map were plotted on graph in C1 and C2. (**D**) Quantification of apparent Young’s Modulus at cell junctions, cell centers and following Latrunculin A treatment (LatA) for hESC-RPE epitheliums and ARPE-19 cell cultures. 21–23 cells were analyzed per condition. Values plotted are mean ± SEM. Significant Kruskal–Wallis tests were followed by Dunn’s multiple comparisons tests. *p < 0.05; **p < 0.01; ***p < 0.001; ****p < 0.0001

Actin microfilaments play an essential role in mechanotransduction and in shaping cell architecture as a response to force signals [31]. Hence, the structure of actin assemblies has a direct effect on cell stiffness [32]. Force-indentation experiments revealed differences in the structure and mechanical properties of hESC-RPE and ARPE-19 cells (Fig. 5). hESC-RPE epitheliums exhibited a heterogeneous distribution of stiffness, with cell junctions being often stiffer than cell centers (Fig. 5A and C). In ARPE-19 cells, a dense network of filamentous structures formed nodes and bundles with high stiffness (up to 50 kPa). These mechanical observations are consistent with the actin distribution observed using phalloidin labelling (Fig. 2). To estimate the effect of destabilizing actin cortical networks on apical membrane stiffness of RPE cells, we treated hESC-RPE and ARPE-19 cells with Latrunculin A (LatA, 1mM), just after a 2-week culture period allowing epithelial formation (Fig. 5A and B). LatA induced a significant lowering of the cell stiffness on the apical side of living RPE cells for both lines. Areas of high stiffness in hESC-RPE epitheliums (cell borders) and ARPE-19 cells (filaments and bundles) disappeared; this supports the view that actin networks drive cell stiffness in our experiments.

To gain insights into the tightening of cell junctions, we compared cell stiffness at the junctions with cell stiffness at the centers of cells for both hESC-RPE and ARPE-19 cells. Although some junctions were not stiffer than cell centers (Fig. 5C and C2), the mean stiffness was significantly higher at cell junctions than in cell centers for both ARPE-19 and hESC-RPE cells (Fig. 5D). Whereas this confirms RPE epithelial formation, it also suggests that the monolayer can display heterogeneity in the stiffness of junctions.

### High-resolution Topographic Images Reveal Actin-based Protrusions on Apical Membrane of RPE Epitheliums

**Fig. 6:**
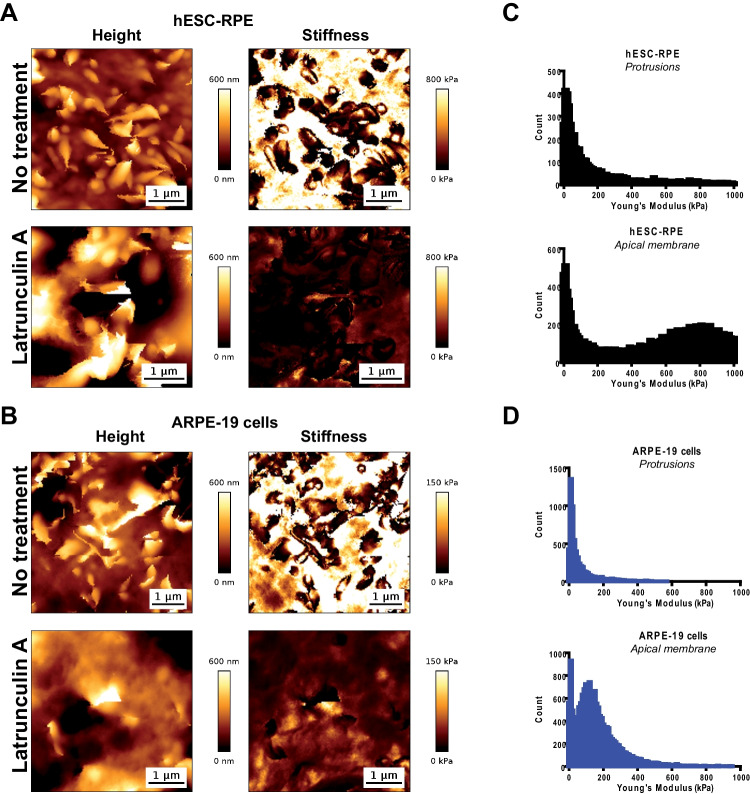
High-resolution AFM topographical images and corresponding elasticity maps revealed typical microvilli in the apical membrane of RPE cells. (**A–B**) AFM topographical images (height) and corresponding elasticity maps (stiffness) of hESC-RPE epitheliums (**A**) and ARPE-19 cells (**B**) following a 2-week culture period. Cells were analyzed using an AFM without treatment or after Latrunculin A exposition. (**C–D**) Histograms of the distribution of Young’s modulus at the apical membrane, obtained from elasticity maps at a threshold of 0.05 µm height. hESC-RPE (**C**) and ARPE-19 (**D**) Protrusion histograms correspond to apparent Young’s modulus distribution at height higher than this threshold. hESC-RPE (**C**) and ARPE-19 (**D**) Apical membrane histograms corresponds to apparent Young’s modulus below this threshold. Data are obtained following fixation of the same batches of cells used for live cell AFM analysis in Fig. 5

Next, we fixed cells using paraformaldehyde to image cells at a higher resolution using AFM. Fixing cells implies freezing membranes at the cell surface; as a result, it allowed distinguishing apical protrusions on top of the plasma membrane in topographical images (Fig. 6A-B). These apical protrusions were present in both cell lines (hESC-RPE and ARPE-19 cells). Following LatA treatment, apical protrusions of the membrane almost disappeared from topographical images in both conditions, suggesting an involvement of actin networks in these structures and supporting the hypothesis that we directly observed by AFM the microvilli marked by EZRIN on Fig. 1. However, it should be noted that the sharp horizontal boundaries between dark and light regions in the image of LatA-treated cells (Fig. 6A) are likely to be artifacts caused by residual mobile protrusions displaced by the AFM cantilever. We next quantified the stiffness of the apical membrane surrounding the apical protrusions in fixed cells. As shown both in stiffness maps (Fig. 6B) and in graphs representing the distributions of stiffnesses in apical protrusions vs. apical membranes (Fig. 6C and D), protrusions appeared softer than membranes both in hESC-RPE and ARPE-19 cells. Taken together, these results indicate that apical protrusions reminiscent of microvilli point outwards the cell membrane of RPE cell epithelium; moreover, these structures are supported by actin networks.

## Discussion

In this study, we differentiated three hPSC lines into RPE cells. These cells were then characterized for their ability to form a cohesive epithelium with the polarized expression of characteristic RPE markers. In addition, the obtained RPE epitheliums were fully functional (barrier function assessed by TEER and ability to release cytokines). We then probed for the first time the mechanical properties of hPSC-RPE cells using an AFM. We showed that stiffness of the apical surface of hPSC-RPE cells was inversely correlated with epithelial formation and cell density. Moreover, at a subcellular level, cell–cell junctions in RPE epitheliums appeared stiffer that cell centers. This higher stiffness observed in tight junctions is dependent on actin networks. Finally, we also observed apical protrusions supported by actin networks on top of RPE epitheliums with a lower stiffness compared to the corresponding apical membrane. Taken together, the mechanical parameters that we defined in this study could be used to evaluate the quality of RPE epithelium generated for cell therapy.

RPE cell therapy is a promising approach to prevent photoreceptor degeneration in AMD and in some forms of RP associated with a RPE dysfunction [4]. In particular, the production of RPE cells from hPSCs was developed extensively, with numerous differentiation protocols. This RPE production has recently been extended to reach an industrial scale through automation [33–35]. Our lab and others have improved the formulation of the final cell therapy product to enhance therapeutic outcomes, the RPE sheet formulation being the more potent [21, 36, 37]. Finally, a number of clinical trials demonstrated the safety of these cell therapy products [6–9]. However, RPE sheets are living biological products and the quality controls required to address efficiently the functionality of RPE epitheliums suitable for transplantation (i.e. the final formulation of the cell therapy product) are scarce and time-consuming. We addressed this need by exploring for the first time the nanomechanics of hPSC-RPE sheets. The few studies that addressed so far the mechanical properties of RPE epitheliums used mainly an adult RPE cell line (ARPE-19 cells) as a model, whereas we used hPSC-derived RPE cells. Indeed, Wiktor and collaborators measured similar stiffnesses of ARPE-19 cells using an AFM [12]. In this same study, ARPE-19 cells had a morphology and actin network similar to what we observed. Together with our results, this strongly supports the robustness and reproducibility of stiffness measurements across distinct laboratories.

However, the morphology of ARPE-19 cells is different from the morphology of primary cultures of RPE cells [27, 38]. ARPE-19 cells resemble more closely to RPE cells that have undergone an epithelial-mesenchymal transition with the presence of actin stress fibers [39]. At the opposite side of the spectrum, hPSC-RPE sheets adopt a morphology that resembles that of a native RPE epithelium. Therefore, measuring the stiffness of hPSC-RPE cells is more informative than measuring the stiffness of ARPE-19 cells. Here, we show for the first time that hPSC-RPE have different mechanical properties according to the stage of epithelial formation. hPSC-RPE cell stiffness measured in the center of the cells decreased during the epithelial formation period. In addition, at a subcellular level, epithelial formation was associated with the apparition of tight junctions at the intersection between cells and with an increased cell stiffness at hPSC-RPE cell borders. Therefore, nanomechanics of hPSC-RPE cells informs on the epithelial reformation in vitro. It should be noted that not all individual cell junctions have a higher stiffness compared to the nearest cell center. We speculate that probably not all cells reached a complete maturation stage with tight junctions at the time of analysis (2-week culture). Indeed, it takes several weeks to reach an advanced epithelial maturation with hPSC-RPE cells [5, 27]. Longer culture periods cannot be achieved in our AFM setting; the epithelium had the tendency to detach from the culture dish after 2–3 weeks of culture, which is not the case in culture insert. This is probably due to the strengthening of tight junctions and the absence of a lower compartment. Optimization of culture settings suitable for AFM may increase the culture duration (for example, using other protein coatings of culture dishes or adapting the AFM acquisitions on culture insert). Nevertheless, mean values of stiffness at cell borders were significantly higher compared to cell centers, thus indicating that early detection of epithelial formation can be obtained with AFM measurements.

Functional analysis of RPE sheets revealed differences between the four different lines. Both VEGF secretions and TEER values allowed distinguishing two groups: hiPSC1-RPE and hESC-RPE in one hand; and hiPSC2-RPE and ARPE-19 cells in the other hand. However, these differences were not observed when analyzing cell stiffness data. It suggests that the nanomechanical data provides different types of information, which are complementary to TEER or VEGF measures to characterize the cell therapy product. Conversely, VEGF and TEER provide a similar characterization and we could speculate that only one of these may be required as a quality control.

Upon maturation, RPE cells adopt an apico-basal polarity with the formation of microvilli. These membrane protrusions are usually observed by electron microscopy, which requires long preparation of the samples. At the opposite, using only the AFM and a simple fixation process (15 min of PFA treatment) in a label-free approach, we were able to visualize microvilli. Microvilli were not detectable on live cells. It suggests that these structures are either too soft or too mobile to be detected using our AFM settings. PFA fixation increased the apparent stiffness, thus revealing microvilli in topography maps. It should be noted that the inner mobility of microvilli might also influence the measurement of apparent stiffness in fixed cells. Indeed, there is a risk that these protrusions bend under the cantilever, contributing to the much lower apparent stiffness. In this study, we considered only the relative apparent stiffness, as they are useful to highlight the contrast between the microvilli and the underlying cell surface. Such microvilli were also observed using AFM on fixed adult primary human RPE cells [39] or in other cellular contexts [40–42]. Microvilli structures depend on actin microfilaments rather than microtubules. Indeed, destabilization of actin networks altered microvilli as observed by AFM and electron microscopy and decreased cell stiffness in Hela cells [43]. At the opposite, destabilization of microtubules did not affect neither microvilli nor mechanical properties of these cells. Thus, our results following actin destabilization confirmed that we actually observed microvilli on AFM structural images and that cell stiffness depends also on actin networks in the context of RPE cells.

In the ARPE-19 cell model, treatments with lipofuscin and blue light irradiation induced a sub-lethal stress or weakly lethal stress [12]. As a result, the cell cytoskeleton was disrupted and cell stiffness decreased. Similar observations were done on other cellular models [15] or with other types of lethal stress [11]. At the opposite, treatment of ARPE-19 cells with a ROCK inhibitor increased cell stiffness and reduced apoptosis [13]. Thus, probing cell stiffness in RPE cells may inform on the global health of the cell culture prior to cell death. In this study, we did not evaluate the impact of sub-lethal or lethal stress on the nanomechanical properties of hPSC-RPE epitheliums. We only evaluated the impact of cytoskeletal destabilization on cell stiffness. Future studies may be required to measure elasticity on hPSC-RPE epitheliums after triggering a sub-lethal or weekly lethal stress. It will allow specifying a range of acceptable values of mean elasticity to set up a quality control of the global health of hPSC-RPE epitheliums.

## Conclusion

In this study, we demonstrated that label-free nanomechanical measurements on hPSC-RPE sheets provide information regarding epithelial formation stage and structural organization of apical membranes. These AFM measurements can be implemented as quality control to evaluate the suitability of RPE sheets produced for cell therapy. Information collected with AFM is complementary and different from other standard quality controls (cytokine release, TEER measurements). The exploration of new approaches to characterize cell therapy products in a simple manner, in particular the ones that are formulated as a tissue or a sheet is essential for the success of cell therapy. Indeed, a better understanding of the biology and/or the physiological state (including normal range of physiological variations) of a biological product will allow predicting the ones that are suitable for transplantation and ultimately will potentiate in vivo functionality. Therefore, such AFM mechanical studies can be extended to other cell therapy products to improve their therapeutic outcomes in vivo.

## Experimental Procedures

### Cell Culture

The clinical grade hESC line RC-9 (referred as hESC) has been derived by Roslin Cells (Edinburgh, Scotland) and was described in our previous publication [21] and in [44]. hiPSC-GFP (referred as hiPSC1) was obtained from Coriell Institute (USA; AICS-0036 cell line). hiPSC PC-1432 (referred as hiPSC2) was reprogramed (OSKM episomes) by Phenocell (Grasse, France) and was already described [45]. hPSCs were cultured with Stempro hESC medium (Thermo Fischer scientific, USA) supplemented with FGF2 (Miltenyi Biotec, Germany). Culture dishes were coated with L7 hPSC matrix (Lonza Bioscience, USA) and culture medium was change 3 times a week. hPSC lines were controlled by m-FISH for genetic stability (data not shown).

RPE cells were differentiated from hPSCs according to our previously published protocol [21]. Briefly, the differentiation medium was composed of Dulbecco’s modified Eagle’s medium (high glucose, Thermo Fisher Scientific, USA) with 20% knockout serum replacement (KSR, Thermo Fisher Scientific), supplemented with 50 μM β-mercaptoethanol and 1% nonessential amino acids supplement (Thermo Fisher Scientific, USA). KSR concentration was then reduced to 4% following pigmented patches manual isolation and amplification. Culture dishes were coated with L7 hPSC matrix (Lonza Bioscience, USA) all along the differentiation process. Cells were detached and dissociated with TrypLE during the amplification process (Thermo Fisher Scientific, USA). hPSC-RPE cells were banked at passage 1 (P1) in Cryostor CS10 medium (BioLife Solutions Inc., USA) and stored in liquid nitrogen. hPSC-RPE cells were thawed at 500 000 cells per cm^2^ for the different experiments. ARPE-19 cells (CRL-2302 cell line from ATCC, USA) correspond to an adult RPE cell line and were cultured with Dulbecco's modified Eagle’s medium: nutrient mixture F-12 (DMEM/F12) supplemented with 10% fetal bovine serum (Thermo Fischer scientific, USA). When indicated in the text, Latrunculin A (L12370, Thermo Fisher Scientific, USA) was applied at 1µM at least one hour on hPSC-RPE and ARPE-19 cells cultured for 14 days.

### VEGF ELISA Assay

hPSC-RPE and ARPE-19 cells were grown on Transwell membrane (Corning) coated with L7™ hPSC matrix (Lonza Bioscience, USA). Culture media from apical and basal compartments were collected every week. VEGF measurements were performed using the human VEGF Quantikine ELISA kit (R&D System, USA) according to manufacturer instruction.

### Immunostaining and Image Acquisition

Immunofluorescence staining was performed on hPSC-RPE and ARPE-19 cells cultured on Transwell membrane (Corning, USA). Cells were blocked with 10% normal goat serum (NGS) in PBS 0.1% triton × 100. Primary antibodies used in this study were MERTK (ab5968, Abcam, UK) and EZRIN (E8897, Merck, Germany). Cells were incubated overnight at 4°C with primary antibodies diluted in PBS 0.1% triton X100 with 5% NGS. Specific antibodies coupled to Alexa Fluor 488 and 555 were used as secondary antibodies. Phalloidin-AF647 (A22287, Thermo Fischer scientific, USA) was incubated at the same time as secondary antibodies. Nuclei were counterstained with either DAPI (Cell Signaling) and mounted in Fluoromount G (Southern Biotech, USA). Images were acquired with an inverted confocal microscope (Zeiss). For zx images, xy stacks covering cell width were resliced in zx then followed by a maximal projection using ImageJ software. For xy images, a maximal projection was realized around cell width.

### Measurement of Transepithelial Electrical Resistance

hPSC-RPE and ARPE-19 cells were cultured on transwell membranes. TEER was measured using an EVOM2 epithelial Voltohmmeter (World Precision Instruments, USA) with STX2 hand held electrodes. To obtain TEER values of a target sample, output value of the blank (transwell insert without cells) was subtracted from the sample output. Values are expressed as ohm.cm^2^.

### Atomic Force Microscopy

hPSC-RPE and ARPE-19 cells were cultured in 6 cm of diameter culture dishes. Topography and mechanical mapping was obtained using the Quantitative Imaging™ (QI) mode of the Nanowizard 4 Bioscience AFM from JPK/Bruker. In QI mode, the AFM tip is driven vertically to collect a force-distance curve for each pixel of the image. During the downwards approach, the AFM tip contacts the cell and indents it. When it reaches the force setpoint, that is, the maximum force applied by the tip to the sample (300–600 pN in our experiments), the AFM tip moves upwards and retract from the cell, before moving laterally and starting a new approach-retract cycle corresponding to a new pixel. We used PeakForce QNM-Live Cell cantilevers (PFQNM-LC-A-CAL; Bruker AFM Probes) that have triangular pyramid shape, half-opening angle of 15°, and a rounded end of 65 nm radius [42]. In all experiments, cantilever sensitivity was obtained by collecting a force curve on a stiff glass surface. The spring constant of the cantilever was then calibrated using the thermal tune method. As recommended for the calibration of this type of cantilever, the sensitivity of the cantilever and thermal spectrum collection were performed in two independent steps [46]. The spring constant was in a range of 0.06–0.10 N/m. Force-indentation curves were collected with 100 μm/s probe velocity over a variable area (10–100 μm2) with a resolution of 64 × 64 and up to 256 × 256 pixels. AFM experiments were performed in liquid at 30°C under air flow containing 5% CO2 and within 2 h after the cells were taken out of the incubator. In some experiments, cells were fixed using 4% formaldehyde for 30 min at room temperature and rinsed with PBS before AFM imaging.

### Analysis of AFM Force-Distance Curves

To quantify the cell stiffness, we calculated the Young’s modulus E by fitting a Hertz-Sneddon model to the force-distance curves using the Bilodeau formula for indentation with a triangular pyramid [47]: F = 0.8887 E/(1-v^2^) tanα δ^2, where F is the measured force, α the half opening angle of the tip, v the Poisson ratio, assumed to be 0.5 in our experiments, and δ the indentation (Fig. S1). We use that model that assumes a pyramid shape for the contact geometry because in our experiments on living cells, maximum indentations are much larger than the radius of the AFM tip. In the case of fixed cells that are much stiffer than living cells, the maximum indentation is in the order of the tip radius. As a result, using the pyramid shape approximation leads to underestimating the contact area and thus should overestimate the Young’s modulus of fixed cells. As in our study we first and foremost wanted to highlight stiffness contrasts to make microvilli more visible, we kept the same model for contact geometry despite its obvious limitations. Curve fitting was performed using the JPK Data Processing software (version 6.3.49). Fitting a Hertz-Sneddon model to the data provides the location of the contact point, that is, the altitude at which the AFM tip contacts the cell. We used the Hertz-Sneddon model rather that the JKR model as we found no sign of adhesion forces at the vicinity of the contact point (Fig. S1). In addition, we also collected topography maps in which each pixel corresponds to the precise vertical position the tip has at a specific force, that we refer to as height at (a given force) maps. These maps better reflect the structural details present inside the cell, as they exhibit better contrast between soft cytoplasmic areas and stiffer cytoskeletal filaments such as actin stress cables, for instance. In this paper, the AFM images in Fig. 4C show the contact point height. All other AFM images display the height at ~ 300 pN. Indentation depth was in the order of 500 nm (cell centers) vs. 300 nm (cell junctions) for live cells and of 40 nm (cell surface) vs. 80 nm for fixed cells. Some ARPE-19 cell junctions had a higher stiffness resulting in a lower indentation depth. In our setup and within an indentation speed range of 1–100 µm/sec, we believe viscoelastic effects had no significant impact on the mechanical response of our samples (regarding Young’s modulus) as we saw no effect on other living cells that have a Young’s modulus in the kilopascal range [46].

### Statistical Analysis

GraphPad Prism 9.3.1 (GraphPad Software, Inc., USA) was used for statistical analysis. Significant two-way ANOVAs were followed up with Bonferroni posttest. Mann Whitney test was used when two groups unpaired were analyzed. When more than two groups, Krukal-Wallis non-parametric test was used followed by Dunn’s multiple comparisons test; *p < 0.05, **p < 0.01, and ***p < 0.001. Complete statistical analysis is included in supplementary materials.

### Supplementary Information

Below is the link to the electronic supplementary material.

Supplementary file1 (DOCX 98 KB)

## Data Availability

The materials and data that support the findings of this study are available from corresponding authors upon reasonable request.
