# Supplementary material for: Biomechanical Characterization of Retinal Pigment Epitheliums Derived from hPSCs Using Atomic Force Microscopy
Source: Stem Cell Rev Rep. 2024 Apr 16;20(5):1340–52. doi: 10.1007/s12015-024-10717-3 (PMC11222240; doi:10.1007/s12015-024-10717-3)
Supplement: Supplementary file 1 — Supplementary file1 (DOCX 98 KB) [file 12015_2024_10717_MOESM1_ESM.docx]

**Supplementary information**

**Biomechanical characterization of retinal pigment epitheliums derived from hPSCs using atomic force microscopy**

Elise Herardot^1^, Maxime Liboz^2^, Guillaume Lamour^2^, Michel Malo^2^, Alexandra Plancheron^1,3^, Walter Habeler^1,3^, Camille Geiger^1,3^ , Elie Frank^1^, Clément Campillo^2,4^, Christelle Monville^1*^ and Karim Ben M’Barek^1,3*^

*^1^Université Paris-Saclay, Université d’Evry, INSERM UMR861, I-Stem, AFM, 91100 Corbeil-Essonnes, France;*

*^2^Université Paris-Saclay, Univ Evry, CY Cergy Paris Université, CNRS, LAMBE, 91025, Evry-Courcouronnes, France;*

*^3^Centre d’Etude des Cellules Souches, AFM, 91100 Corbeil-Essonnes, France;*

*^4^ Institut Universitaire de France (IUF).*

*Correspondence: [cmonville@istem.fr](mailto:cmonville@istem.fr) and [kbenmbarek@istem.fr](mailto:kbenmbarek@istem.fr)

**Supplementary Figure S1:**


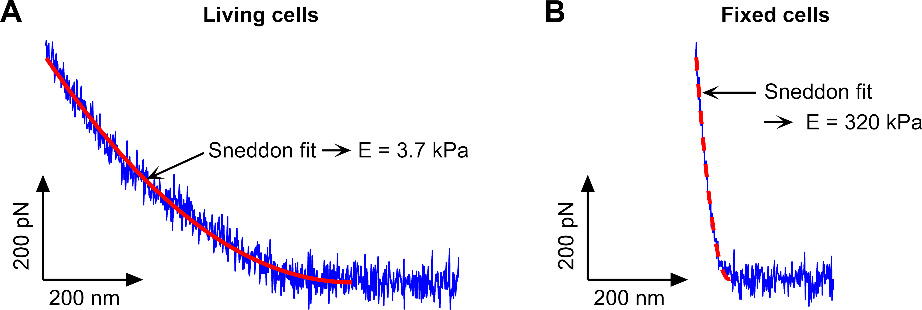


**Legend of supplementary Figure S1**:

Representative force distance curves collected on (A) living RPE cells and on (B) fixed RPE cells. The red lines represent least-square fits of the data using a Sneddon model to extract the Young’s moduli E from the indentation segments of the force curves.

**Detailed statistical analysis:**

**Figure 3B:**

| Table Analyzed | Data 1 |  |  |  |
| --- | --- | --- | --- | --- |
|  |  |  |  |  |
| Two-way RM ANOVA | Matching by cols |  |  |  |
|  |  |  |  |  |
| Source of Variation | % of total variation | P value |  |  |
| Interaction | 16,48 | < 0.0001 |  |  |
| Time | 65,68 | < 0.0001 |  |  |
| rpe line | 13,64 | < 0.0001 |  |  |
| Subjects (matching) | 1,1037 | 0,2237 |  |  |
|  |  |  |  |  |
| Source of Variation | P value summary | Significant? |  |  |
| Interaction | *** | Yes |  |  |
| Time | *** | Yes |  |  |
| rpe line | *** | Yes |  |  |
| Subjects (matching) | ns | No |  |  |
|  |  |  |  |  |
| Source of Variation | Df | Sum-of-squares | Mean square | F |
| Interaction | 12 | 12000 | 1000 | 14 |
| Time | 4 | 48000 | 12000 | 170 |
| rpe line | 3 | 10000 | 3300 | 33 |
| Subjects (matching) | 8 | 810 | 100 | 1,4 |
| Residual | 32 | 2300 | 71 |  |
|  |  |  |  |  |
| Number of missing values | 0 |  |  |  |
|  |  |  |  |  |
| Bonferroni posttests |  |  |  |  |
|  |  |  |  |  |
| hESC-RPE vs hiPSC2-RPE |  |  |  |  |
| rpe line | hESC-RPE | hiPSC2-RPE | Difference | 95% CI of diff. |
| 2,0 | 0,00 | 0,00 | 0,00 | -24 to 24 |
| 9,0 | 19 | 17 | -1,2 | -25 to 23 |
| 23 | 49 | 19 | -30 | -54 to -5.6 |
| 30 | 81 | 26 | -55 | -79 to -31 |
| 37 | 120 | 54 | -61 | -86 to -37 |
|  |  |  |  |  |
| rpe line | Difference | t | P value | Summary |
| 2,0 | 0,00 | 0,00 | P > 0.05 | ns |
| 9,0 | -1,2 | 0,17 | P > 0.05 | ns |
| 23 | -30 | 4,2 | P<0.001 | *** |
| 30 | -55 | 7,7 | P<0.001 | *** |
| 37 | -61 | 8,6 | P<0.001 | *** |
|  |  |  |  |  |
| hESC-RPE vs hiPSC1-RPE |  |  |  |  |
| rpe line | hESC-RPE | hiPSC1-RPE | Difference | 95% CI of diff. |
| 2,0 | 0,00 | 1,8 | 1,8 | -22 to 26 |
| 9,0 | 19 | 18 | -0,60 | -25 to 24 |
| 23 | 49 | 63 | 14 | -9.7 to 39 |
| 30 | 81 | 78 | -3,3 | -27 to 21 |
| 37 | 120 | 110 | -1,5 | -26 to 23 |
|  |  |  |  |  |
| rpe line | Difference | t | P value | Summary |
| 2,0 | 1,8 | 0,25 | P > 0.05 | ns |
| 9,0 | -0,60 | 0,084 | P > 0.05 | ns |
| 23 | 14 | 2,0 | P > 0.05 | ns |
| 30 | -3,3 | 0,46 | P > 0.05 | ns |
| 37 | -1,5 | 0,21 | P > 0.05 | ns |
|  |  |  |  |  |
| hESC-RPE vs ARPE-19 |  |  |  |  |
| rpe line | hESC-RPE | ARPE-19 | Difference | 95% CI of diff. |
| 2,0 | 0,00 | 3,9 | 3,9 | -20 to 28 |
| 9,0 | 19 | 44 | 25 | 0.79 to 49 |
| 23 | 49 | 32 | -16 | -40 to 7.9 |
| 30 | 81 | 48 | -33 | -57 to -9.2 |
| 37 | 120 | 54 | -61 | -86 to -37 |
|  |  |  |  |  |
| rpe line | Difference | t | P value | Summary |
| 2,0 | 3,9 | 0,55 | P > 0.05 | ns |
| 9,0 | 25 | 3,5 | P<0.01 | ** |
| 23 | -16 | 2,3 | P > 0.05 | ns |
| 30 | -33 | 4,7 | P<0.001 | *** |
| 37 | -61 | 8,6 | P<0.001 | *** |
|  |  |  |  |  |
| hiPSC2-RPE vs hiPSC1-RPE |  |  |  |  |
| rpe line | hiPSC1-RPE | hiPSC2-RPE | Difference | 95% CI of diff. |
| 2,0 | 0,00 | 1,8 | 1,8 | -22 to 26 |
| 9,0 | 17 | 18 | 0,60 | -24 to 25 |
| 23 | 19 | 63 | 44 | 20 to 68 |
| 30 | 26 | 78 | 52 | 27 to 76 |
| 37 | 54 | 110 | 60 | 36 to 84 |
|  |  |  |  |  |
| rpe line | Difference | t | P value | Summary |
| 2,0 | 1,8 | 0,25 | P > 0.05 | ns |
| 9,0 | 0,60 | 0,084 | P > 0.05 | ns |
| 23 | 44 | 6,2 | P<0.001 | *** |
| 30 | 52 | 7,2 | P<0.001 | *** |
| 37 | 60 | 8,4 | P<0.001 | *** |
|  |  |  |  |  |
| hiPSC1-RPE vs ARPE-19 p36 |  |  |  |  |
| rpe line | hiPSC1-RPE | ARPE-19 | Difference | 95% CI of diff. |
| 2,0 | 0,00 | 3,9 | 3,9 | -20 to 28 |
| 9,0 | 17 | 44 | 26 | 2.0 to 50 |
| 23 | 19 | 32 | 14 | -11 to 38 |
| 30 | 26 | 48 | 22 | -2.5 to 46 |
| 37 | 54 | 54 | 0,0000038 | -24 to 24 |
|  |  |  |  |  |
| rpe line | Difference | t | P value | Summary |
| 2,0 | 3,9 | 0,55 | P > 0.05 | ns |
| 9,0 | 26 | 3,7 | P<0.01 | ** |
| 23 | 14 | 1,9 | P > 0.05 | ns |
| 30 | 22 | 3,0 | P < 0.05 | * |
| 37 | 0,0000038 | 0,00000053 | P > 0.05 | ns |
|  |  |  |  |  |
| hiPSC2-RPE vs ARPE-19 |  |  |  |  |
| rpe line | hiPSC2-RPE | ARPE-19 | Difference | 95% CI of diff. |
| 2,0 | 1,8 | 3,9 | 2,1 | -22 to 26 |
| 9,0 | 18 | 44 | 26 | 1.4 to 50 |
| 23 | 63 | 32 | -31 | -55 to -6.5 |
| 30 | 78 | 48 | -30 | -54 to -5.9 |
| 37 | 110 | 54 | -60 | -84 to -36 |
|  |  |  |  |  |
| rpe line | Difference | t | P value | Summary |
| 2,0 | 2,1 | 0,29 | P > 0.05 | ns |
| 9,0 | 26 | 3,6 | P<0.01 | ** |
| 23 | -31 | 4,3 | P<0.001 | *** |
| 30 | -30 | 4,2 | P<0.001 | *** |
| 37 | -60 | 8,4 | P<0.001 | *** |
|  |  |  |  |  |
|  |  |  |  |  |

**Figure 3C:**

| Table Analyzed | **hESC-RPE** |  | |  |  |
| --- | --- | --- | --- | --- | --- |
|  |  |  |  | |  |
| Two-way ANOVA |  |  |  | |  |
|  |  |  |  | |  |
| Source of Variation | % of total variation | P value |  | |  |
| Interaction | 1,69 | 0,0005 |  | |  |
| Column Factor | 1,34 | 0,0001 |  | |  |
| Row Factor | 96,26 | < 0.0001 |  | |  |
|  |  |  |  | |  |
| Source of Variation | P value summary | Significant? |  | |  |
| Interaction | *** | Yes |  | |  |
| Column Factor | *** | Yes |  | |  |
| Row Factor | *** | Yes |  | |  |
|  |  |  |  | |  |
| Source of Variation | Df | Sum-of-squares | Mean square | | F |
| Interaction | 3 | 1764000 | 588000 | | 10,71 |
| Column Factor | 1 | 1399000 | 1399000 | | 25,49 |
| Row Factor | 3 | 100300000 | 33430000 | | 609,0 |
| Residual | 15 | 823500 | 54900 | |  |
|  |  |  |  | |  |
| Number of missing values | 1 |  |  | |  |
|  |  |  |  | |  |
| Bonferroni posttests |  |  |  | |  |
|  |  |  |  | |  |
| Apical vs Basal |  |  |  | |  |
| Row Factor | Apical | Basal | Difference | | 95% CI of diff. |
| D2 | 13,04 | 204,7 | 191,7 | | -351.0 to 734.3 |
| D9 | 552,1 | 583,6 | 31,50 | | -511.2 to 574.2 |
| D16 | 2734 | 3083 | 349,6 | | -257.1 to 956.4 |
| D23 | 4537 | 5956 | 1418 | | 875.7 to 1961 |
|  |  |  |  | |  |
| Row Factor | Difference | t | P value | | Summary |
| D2 | 191,7 | 1,002 | P > 0.05 | | ns |
| D9 | 31,50 | 0,1647 | P > 0.05 | | ns |
| D16 | 349,6 | 1,635 | P > 0.05 | | ns |
| D23 | 1418 | 7,414 | P<0.001 | | *** |
|  |  |  |  | |  |

| Table Analyzed | **hiPSC1-RPE** |  |  |  |
| --- | --- | --- | --- | --- |
|  |  |  |  |  |
| Two-way ANOVA |  |  |  |  |
|  |  |  |  |  |
| Source of Variation | % of total variation | P value |  |  |
| Interaction | 0,89 | 0,0240 |  |  |
| Column Factor | 1,09 | 0,0013 |  |  |
| Row Factor | 96,87 | < 0.0001 |  |  |
|  |  |  |  |  |
| Source of Variation | P value summary | Significant? |  |  |
| Interaction | * | Yes |  |  |
| Column Factor | ** | Yes |  |  |
| Row Factor | *** | Yes |  |  |
|  |  |  |  |  |
| Source of Variation | Df | Sum-of-squares | Mean square | F |
| Interaction | 3 | 1084000 | 361300 | 4,130 |
| Column Factor | 1 | 1324000 | 1324000 | 15,14 |
| Row Factor | 3 | 117800000 | 39250000 | 448,7 |
| Residual | 16 | 1400000 | 87480 |  |
|  |  |  |  |  |
| Number of missing values | 0 |  |  |  |
|  |  |  |  |  |
| Bonferroni posttests |  |  |  |  |
|  |  |  |  |  |
| Apical vs Basal |  |  |  |  |
| Row Factor | Apical | Basal | Difference | 95% CI of diff. |
| D2 | 152,5 | 204,7 | 52,20 | -627.2 to 731.6 |
| D9 | 1214 | 1512 | 297,9 | -381.5 to 977.3 |
| D16 | 4131 | 4481 | 349,2 | -330.2 to 1029 |
| D23 | 5118 | 6298 | 1180 | 500.5 to 1859 |
|  |  |  |  |  |
| Row Factor | Difference | t | P value | Summary |
| D2 | 52,20 | 0,2161 | P > 0.05 | ns |
| D9 | 297,9 | 1,234 | P > 0.05 | ns |
| D16 | 349,2 | 1,446 | P > 0.05 | ns |
| D23 | 1180 | 4,886 | P<0.001 | *** |

| Table Analyzed | **hiPSC2-RPE** |  |  |  |
| --- | --- | --- | --- | --- |
|  |  |  |  |  |
| Two-way ANOVA |  |  |  |  |
|  |  |  |  |  |
| Source of Variation | % of total variation | P value |  |  |
| Interaction | 13,93 | 0,0774 |  |  |
| Column Factor | 18,98 | 0,0044 |  |  |
| Row Factor | 47,42 | 0,0011 |  |  |
|  |  |  |  |  |
| Source of Variation | P value summary | Significant? |  |  |
| Interaction | ns | No |  |  |
| Column Factor | ** | Yes |  |  |
| Row Factor | ** | Yes |  |  |
|  |  |  |  |  |
| Source of Variation | Df | Sum-of-squares | Mean square | F |
| Interaction | 3 | 3489000 | 1163000 | 2,818 |
| Column Factor | 1 | 4755000 | 4755000 | 11,52 |
| Row Factor | 3 | 11880000 | 3959000 | 9,593 |
| Residual | 14 | 5778000 | 412700 |  |
|  |  |  |  |  |
| Number of missing values | 2 |  |  |  |
|  |  |  |  |  |
| Bonferroni posttests |  |  |  |  |
|  |  |  |  |  |
| Apical vs Basal |  |  |  |  |
| Row Factor | Apical | Basal | Difference | 95% CI of diff. |
| D2 | 56,21 | 137,6 | 81,36 | -1421 to 1584 |
| D9 | 206,3 | 409,3 | 203,0 | -1299 to 1705 |
| D16 | 505,8 | 1935 | 1429 | -73.12 to 2931 |
| D23 | 1034 | 3097 | 2063 | 223.3 to 3903 |
|  |  |  |  |  |
| Row Factor | Difference | t | P value | Summary |
| D2 | 81,36 | 0,1551 | P > 0.05 | ns |
| D9 | 203,0 | 0,3869 | P > 0.05 | ns |
| D16 | 1429 | 2,725 | P > 0.05 | ns |
| D23 | 2063 | 3,212 | P < 0.05 | * |
|  |  |  |  |  |

| Table Analyzed | **ARPE-19** |  |  |  |
| --- | --- | --- | --- | --- |
|  |  |  |  |  |
| Two-way ANOVA |  |  |  |  |
|  |  |  |  |  |
| Source of Variation | % of total variation | P value |  |  |
| Interaction | 10,06 | 0,1465 |  |  |
| Column Factor | 3,14 | 0,1841 |  |  |
| Row Factor | 60,68 | 0,0002 |  |  |
|  |  |  |  |  |
| Source of Variation | P value summary | Significant? |  |  |
| Interaction | ns | No |  |  |
| Column Factor | ns | No |  |  |
| Row Factor | *** | Yes |  |  |
|  |  |  |  |  |
| Source of Variation | Df | Sum-of-squares | Mean square | F |
| Interaction | 3 | 1590000 | 530000 | 2,056 |
| Column Factor | 1 | 496900 | 496900 | 1,927 |
| Row Factor | 3 | 9588000 | 3196000 | 12,40 |
| Residual | 16 | 4125000 | 257800 |  |
|  |  |  |  |  |
| Number of missing values | 0 |  |  |  |
|  |  |  |  |  |
| Bonferroni posttests |  |  |  |  |
|  |  |  |  |  |
| Apical vs Basal |  |  |  |  |
| Row Factor | Apical | Basal | Difference | 95% CI of diff. |
| D2 | 433,3 | 408,1 | -25,20 | -1191 to 1141 |
| D9 | 904,9 | 580,9 | -324,0 | -1490 to 842.2 |
| D16 | 1605 | 1898 | 293,4 | -872.8 to 1460 |
| D23 | 2438 | 1343 | -1095 | -2262 to 70.95 |
|  |  |  |  |  |
| Row Factor | Difference | t | P value | Summary |
| D2 | -25,20 | 0,06078 | P > 0.05 | ns |
| D9 | -324,0 | 0,7815 | P > 0.05 | ns |
| D16 | 293,4 | 0,7077 | P > 0.05 | ns |
| D23 | -1095 | 2,642 | P > 0.05 | ns |
|  |  |  |  |  |

**Figure 4B:**

| Table Analyzed | **hiPSC1-RPE** |
| --- | --- |
|  |  |
| Column F | hiPSC1-RPE J16 |
| vs. | vs, |
| Column E | hiPSC1-RPE J2 |
|  |  |
| Mann Whitney test |  |
| P value | <0,0001 |
| Exact or approximate P value? | Exact |
| P value summary | **** |
| Significantly different (P < 0.05)? | Yes |
| One- or two-tailed P value? | Two-tailed |
| Sum of ranks in column E,F | 185 , 46 |
| Mann-Whitney U | 1 |
|  |  |
| Difference between medians |  |
| Median of column E | 4,590, n=12 |
| Median of column F | 1,510, n=9 |
| Difference: Actual | -3,080 |
| Difference: Hodges-Lehmann | -3,230 |

| Table Analyzed | **hiPSC2-RPE** |
| --- | --- |
|  |  |
| Column B | hiPSC2-RPE J16 |
| vs. | vs, |
| Column A | hiPSC2-RPE J2 |
|  |  |
| Mann Whitney test |  |
| P value | 0,0125 |
| Exact or approximate P value? | Exact |
| P value summary | * |
| Significantly different (P < 0.05)? | Yes |
| One- or two-tailed P value? | Two-tailed |
| Sum of ranks in column A,B | 148 , 62 |
| Mann-Whitney U | 17 |
|  |  |
| Difference between medians |  |
| Median of column A | 5,560, n=11 |
| Median of column B | 2,610, n=9 |
| Difference: Actual | -2,950 |
| Difference: Hodges-Lehmann | -3,200 |
|  |  |

| Table Analyzed | **hESC-RPE** |
| --- | --- |
|  |  |
| Column D | hESC-RPE J16 |
| vs. | vs, |
| Column C | hESC-RPE J2 |
|  |  |
| Mann Whitney test |  |
| P value | 0,0004 |
| Exact or approximate P value? | Exact |
| P value summary | *** |
| Significantly different (P < 0.05)? | Yes |
| One- or two-tailed P value? | Two-tailed |
| Sum of ranks in column C,D | 140 , 50 |
| Mann-Whitney U | 5 |
|  |  |
| Difference between medians |  |
| Median of column C | 11,70, n=10 |
| Median of column D | 1,540, n=9 |
| Difference: Actual | -10,16 |
| Difference: Hodges-Lehmann | -8,465 |
|  |  |
| Table Analyzed | **Pool of the 3 RPE lines** |
|  |  |
|  |  |
| Column B | hPSC-RPE J16 |
| vs. | vs, |
| Column A | hPSC-RPE J2 |
|  |  |
| Mann Whitney test |  |
| P value | <0,0001 |
| Exact or approximate P value? | Exact |
| P value summary | **** |
| Significantly different (P < 0.05)? | Yes |
| One- or two-tailed P value? | Two-tailed |
| Sum of ranks in column A,B | 1367 , 463 |
| Mann-Whitney U | 85 |
|  |  |
| Difference between medians |  |
| Median of column A | 6,230, n=33 |
| Median of column B | 1,610, n=27 |
| Difference: Actual | -4,620 |
| Difference: Hodges-Lehmann | -4,220 |

**Figure 5D:**

| Kruskal-Wallis test for **hESC-RPE** |  |
| --- | --- |
| P value | <0,0001 |
| Exact or approximate P value? | Approximate |
| P value summary | **** |
| Do the medians vary signif. (P < 0.05)? | Yes |
| Number of groups | 4 |
| Kruskal-Wallis statistic | 65,82 |
|  |  |
| Data summary |  |
| Number of treatments (columns) | 4 |
| Number of values (total) | 84 |

| Number of families | 1 | |  | |  |  |  |  |
| --- | --- | --- | --- | --- | --- | --- | --- | --- |
| Number of comparisons per family | 6 | |  | |  |  |  |  |
| Alpha | 0,05 | |  | |  |  |  |  |
|  |  | |  | |  |  |  |  |
| Dunn's multiple comparisons test | Mean rank diff, | | Significant? | | Summary | Adjusted P Value |  |  |
| hESC-RPE jct vs. hESC-RPE cell center | 26,02 | | Yes | | ** | 0,0033 | A-B |  |
| hESC-RPE jct vs. hESC-RPE latA jct | 38,88 | | Yes | | **** | <0,0001 | A-C |  |
| hESC-RPE jct vs. hESC-RPE latA cell | 59,57 | | Yes | | **** | <0,0001 | A-D |  |
| hESC-RPE cell vs. hESC-RPE latA jct | 12,86 | | No | | ns | 0,5255 | B-C |  |
| hESC-RPE cell vs. hESC-RPE latA cell | 33,55 | | Yes | | **** | <0,0001 | B-D |  |
| hESC-RPE latA jct vs. hESC-RPE latA cell | 20,69 | | Yes | | * | 0,0359 | C-D |  |
|  |  | |  | |  |  |  |  |
| Test details | Mean rank 1 | | Mean rank 2 | | Mean rank diff, | n1 | n2 | Z |
| hESC-RPE jct vs. hESC-RPE cell | 73,62 | | 47,60 | | 26,02 | 21 | 21 | 3,458 |
| hESC-RPE jct vs. hESC-RPE latA jct | 73,62 | | 34,74 | | 38,88 | 21 | 21 | 5,166 |
| hESC-RPE jct vs. hESC-RPE latA cell | 73,62 | | 14,05 | | 59,57 | 21 | 21 | 7,915 |
| hESC-RPE cell vs. hESC-RPE latA jct | 47,60 | | 34,74 | | 12,86 | 21 | 21 | 1,708 |
| hESC-RPE cell vs. hESC-RPE latA cell | 47,60 | | 14,05 | | 33,55 | 21 | 21 | 4,457 |
| hESC-RPE latA jct vs. hESC-RPE latA cell | 34,74 | | 14,05 | | 20,69 | 21 | 21 | 2,749 |
|  |  | |  | |  |  |  |  |
| Kruskal-Wallis test for **ARPE-19** | |  | |  |  |  |  |  |
| P value | | <0,0001 | |  |  |  |  |  |
| Exact or approximate P value? | | Approximate | |  |  |  |  |  |
| P value summary | | **** | |  |  |  |  |  |
| Do the medians vary signif. (P < 0.05)? | | Yes | |  |  |  |  |  |
| Number of groups | | 4 | |  |  |  |  |  |
| Kruskal-Wallis statistic | | 76,50 | |  |  |  |  |  |
|  | |  | |  |  |  |  |  |
| Data summary | |  | |  |  |  |  |  |
| Number of treatments (columns) | | 4 | |  |  |  |  |  |
| Number of values (total) | | 92 | |  |  |  |  |  |

| Number of families | 1 |  |  |  |  |  |
| --- | --- | --- | --- | --- | --- | --- |
| Number of comparisons per family | 6 |  |  |  |  |  |
| Alpha | 0,05 |  |  |  |  |  |
|  |  |  |  |  |  |  |
| Dunn's multiple comparisons test | Mean rank diff, | Significant? | Summary | Adjusted P Value |  |  |
| ARPE19 jct vs. ARPE19 cell | 26,43 | Yes | ** | 0,0047 | E-F |  |
| ARPE19 jct vs. ARPE19 latA jct | 44,00 | Yes | **** | <0,0001 | E-G |  |
| ARPE19 jct vs. ARPE19 latA cell | 66,52 | Yes | **** | <0,0001 | E-H |  |
| ARPE19 cell vs. ARPE19 latA jct | 17,57 | No | ns | 0,1540 | F-G |  |
| ARPE19 cell vs. ARPE19 latA cell | 40,09 | Yes | **** | <0,0001 | F-H |  |
| ARPE19 latA jct vs. ARPE19 latA cell | 22,52 | Yes | * | 0,0254 | G-H |  |
|  |  |  |  |  |  |  |
| Test details | Mean rank 1 | Mean rank 2 | Mean rank diff, | n1 | n2 | Z |
| ARPE19 jct vs. ARPE19 cell | 80,74 | 54,30 | 26,43 | 23 | 23 | 3,358 |
| ARPE19 jct vs. ARPE19 latA jct | 80,74 | 36,74 | 44,00 | 23 | 23 | 5,589 |
| ARPE19 jct vs. ARPE19 latA cell | 80,74 | 14,22 | 66,52 | 23 | 23 | 8,450 |
| ARPE19 cell vs. ARPE19 latA jct | 54,30 | 36,74 | 17,57 | 23 | 23 | 2,231 |
| ARPE19 cell vs. ARPE19 latA cell | 54,30 | 14,22 | 40,09 | 23 | 23 | 5,092 |
| ARPE19 latA jct vs. ARPE19 latA cell | 36,74 | 14,22 | 22,52 | 23 | 23 | 2,861 |
